# Smart Hydrogels for Treatment of Microbial Diseases

**DOI:** 10.3390/pharmaceutics18020198

**Published:** 2026-02-03

**Authors:** Burak Ünlü, Jose Luis Ropero-Vega, Juan Manuel Alvarez-Caballero, Johanna Marcela Flórez-Castillo, Serbülent Türk

**Affiliations:** 1 Department of Chemistry, Faculty of Science, Sakarya University, 54187 Sakarya, Türkiye; burakunlu@sakarya.edu.tr; 2 Biomedical, Magnetic and Semiconductor Materials Application and Research Center (BIMAS-RC), Sakarya University, 54187 Sakarya, Türkiye; 3 Grupo Química y Bioprospección de Productos Naturales, Universidad del Magdalena, Santa Marta 470004, Colombia; jroperolv@unimagdalena.edu.co (J.L.R.-V.); jalvarez@unimagdalena.edu.co (J.M.A.-C.)

**Keywords:** antimicrobial resistance, biofilm disruption, controlled drug delivery, stimuli-responsive hydrogels

## Abstract

Smart hydrogels, which combine hydrogel properties such as biocompatibility, high drug loading capacity, and injectability while being responsive to external stimuli, are a subclass of smart materials. Smart hydrogels respond to effects that are not harmful to the human body, such as temperature, pH, light, and biomolecules. Furthermore, some smart hydrogels possess dual-responsive properties or can be multifunctional, exhibiting both adhesive and responsive behavior to external stimuli. Smart hydrogels have made groundbreaking advances in the field of biomedical. They have been improved through structural modifications and by gaining the ability to be multi-responsive. Controlling drug release and biofilm disruption by using these smart hydrogels is one of the efficient strategies to reduce antimicrobial resistance and the number of deaths caused by microbial diseases. In this review, the preparation of smart hydrogels, their various types and applications in the treatment of microbial diseases were investigated.

## 1. Introduction

One of the most urgent global public health priorities is reducing mortality from infectious diseases, a challenge exacerbated by the growing prevalence of antimicrobial resistance (AMR). In 2019, infection-related deaths were estimated at 13.7 million, of which 7.7 million were associated with antimicrobial-resistant bacterial pathogens. *Escherichia coli*, *Staphylococcus aureus*, *Streptococcus pneumoniae*, *Klebsiella pneumoniae*, and *Pseudomonas aeruginosa* were the leading bacteria in 54.9% of these deaths [1]. AMR not only leads to therapeutic failure and prolonged recovery but also imposes significant economic burdens, including longer hospital stays and greater demand for medical resources [2,3,4,5,6]. This scenario underscores the urgent need for innovative strategies that move beyond conventional antibiotics and adopt a holistic approach—one that integrates pathogen elimination, minimizes side effects, and leverages delivery platforms capable of protecting therapeutic agents, prolonging their half-life, and enabling localized, sustained, and controlled release [7].

Smart materials can reversibly alter their structure or properties in response to external stimuli such as mechanical stress, pH, temperature, electricity, or light. They are used in a wide range of applications, including sensors, actuators, optoelectronic devices, medicine, and controlled drug release [8,9]. Among the most common smart materials for fabricating actuators are shape-memory alloys and piezoelectric materials. However, their operational conditions can be a limiting factor. Alternatively, polymer-based smart materials offer advantages such as flexibility, light weight, and often biocompatibility, but they typically suffer from poor mechanical strength [10,11,12]. Consequently, ongoing research is focused on developing novel methods and materials to overcome these disadvantages.

While the term "hydrogel” was first used in 1900 to define colloidal gels of inorganic salts, Wichterle’s study, published in 1960, is considered the first hydrogel in the literature [13]. The study was focused on poly(2-hydroxyethyl methacrylate), which was synthesized by free radical polymerization [14]. Due to its weak mechanical strength and poor oxygen permeability, N-vinyl pyrrolidone began to be used as a co-monomer with 2-hydroxyethyl methacrylate in the 1970s. These first hydrogels were synthesized by free radical polymerization of water-soluble monomers with crosslinking agents. Following this progress, hydrogels have been evaluated as materials that respond to changes in their surroundings [15]. Hydrogels are materials that contain three-dimensional networks of polymers. Hydrogels are soft materials, and due to their hydrophilic matrices, they contain a very high amount of water. Drugs can be effectively loaded in hydrogels due to their high-water content and can be released easily owing to their swelling/deswelling properties. However, drug release cannot be controlled in traditional hydrogels. Hence, smart polymers have been integrated into the network of hydrogels, enabling these gels to respond to external stimuli. Additionally, stimuli-responsive or functional materials can be incorporated into the hydrogel matrices to make them smart and multifunctional. In this way, there is significant control over the drug release to impede antimicrobial resistance. Additionally, by integrating smart hydrogels as therapeutics in biomedical applications, microbial biofilm formation at wounds can be inhibited, and formed microbial biofilms can be broken with non-harmful stimuli to which smart hydrogels can respond.

## 2. Preparation of Smart Hydrogels

Hydrogels can be prepared by physical or chemical crosslinking methods. Physical crosslinking is based on non-covalent interactions such as hydrogen bonding and ionic interactions. Physical crosslinking is reversible; therefore, sol–gel transitions can occur. In this way, these hydrogels can be injectable. Additionally, physically crosslinked hydrogels exhibit enhanced self-healing properties. These hydrogels exhibit greater biocompatibility due to the non-use of initiators or crosslinking agents that may be harmful to the human body. However, they have low mechanical strength and poor long-term stability.

On the other hand, chemical crosslinking provides a permanent and irreversible network structure to hydrogels. Therefore, hydrogels prepared by chemical crosslinking exhibit mechanical strength and insolubility. These hydrogels also ensure higher thermal stability. However, using initiators to start crosslinking reactions and chemicals to obtain crosslinked networks can cause cytotoxicity. Hence, additional purification may be necessary for use in biomedical applications.

### 2.1. Physical Crosslinking Methods

Preparation of smart hydrogels using physical crosslinking methods includes hydrogen bonds, ionic interactions, hydrophobic associations, van der Waals forces and Freeze–Thaw cycles. These methods can affect the mechanical properties of hydrogels and the responsiveness of smart hydrogels. Additionally, due to the reversible nature of these bindings, the smart hydrogels with physical crosslinking exhibit enhanced self-healing properties. Furthermore, eliminating the use of hazardous chemicals with physical methods makes these smart hydrogels biocompatible.

**Figure 1:**
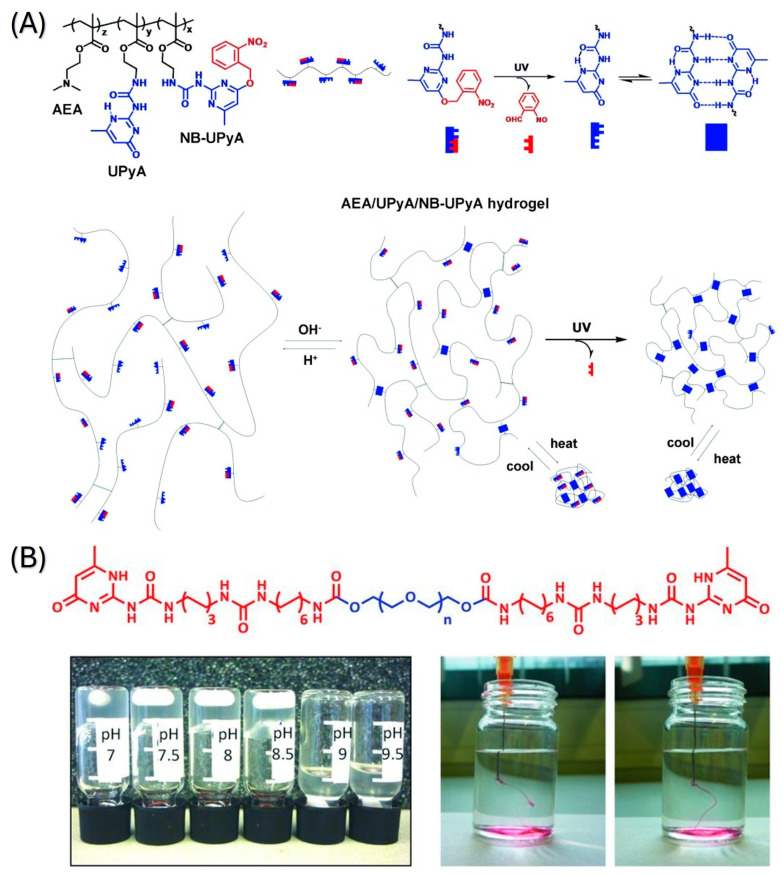
Examples of physical crosslinking by hydrogen bonding, (**A**) Multiresponsive hydrogel system based on UPy and poly [2-(dimethylamino)ethyl methacrylate] [20], (**B**) pH-responsive smart hydrogel based on UPy and poly(ethylene glycol) [21].

Hydrogen bonds are physical interactions between partially positively charged hydrogen atoms with functional groups that contain electron-rich atoms (oxygen, nitrogen, etc.), such as carboxyl, carbonyl or amino groups. While hydrogen bonding can be both intermolecular and intramolecular for crosslinking approaches, intramolecular hydrogen bonding is more critical for preparing hydrogel networks [16]. The single hydrogen bond is a weak force; however, multiple hydrogen bonding formations are stronger and cause ordering in hydrogels [17]. Hence, motifs that can form multiple hydrogen bonds have been utilized [18]. Ureido-pyrimidone (UPy) is an example of self-complementary quadruple hydrogen bonding. It has been widely used for achieving stable structures [19]. UPy-modified polymers can be crosslinked for the formation of hydrogels with multiple responses. One example of UPy usage, as shown in Figure 1A, is poly [2-(dimethylamino)ethyl methacrylate], which has thermal and pH responses with UPy modification [20]. The preparation of the gel was achieved under a nitrogen atmosphere at 60 °C for 1 h. The obtained smart hydrogel showed reversible collapse upon temperature increase, reversible swelling upon pH decrease, and irreversible shrinkage by UV light exposure while maintaining its self-healing property. Another pH-responsive hydrogel, as shown in Figure 1B, was prepared using UPy-coupled poly(ethylene glycol) chains [21]. Hydrogel formation was done by mixing diamine-terminated PEG and UPy-isocyanate at room temperature for 16 h. When the pH was adjusted to a basic region with a threshold at pH 8.5, the hydrogel became fluid. At neutral pH, it reverted to a gel state reversibly.

Another pH-responsive smart hydrogel that is crosslinked by hydrogen bonding [22], which is the poly(vinyl pyrrolidone)/chitosan hydrogel, which was loaded with Zn, ZAg MOF or Ag nanoparticles [23]. PVP/chitosan hydrogel was synthesized by mixing chitosan solution in acetic acid with PVP for 2 h. Gelation of the hydrogel was initiated by adding NaOH, and the obtained hydrogel was dried at 40 °C for 72 h. Yang et al. investigated a dual-responsive, temperature and pH-responsive hydrogel prepared by self-assembly using pseudoephedrine and glycyrrhizic acid, which utilized hydrogen bonding [24]. Pseudoephedrine and glycyrrhizic acid were dissolved and heated to obtain homogenization, followed by cooling to obtain gel form.

Ionic/electrostatic interactions are another physical crosslinking interaction between oppositely charged polymers or between polymers and metal ions. These reversible interactions facilitate rapid self-assembly, providing smart hydrogels with tunable properties [25]. Smart hydrogels formed through ionic interactions are typically responsive to pH and electric fields and also possess self-healing properties [26]. The most common hydrogels of this type are based on alginate, a natural water-soluble polymer. It can be crosslinked by divalent (e.g., Ca^2+^, Cu^2+^) or trivalent (e.g., Fe^3+^, Al^3+^) metal ions [27]. Although alginate forms hydrogels easily via this method, the resulting material is not responsive to external stimuli. Nevertheless, incorporating stimuli-responsive polymers into the alginate network can confer “smart” characteristics to the hydrogel while preserving the high stability inherent to alginate matrices. Roquero et al. modified alginate calcium networks by poly(vinyl alcohol)-diboronate [28]. Due to the reactivity of boronate esters under oxidative stress conditions, the interpenetrating polymer network of the hydrogel was degraded in the presence of low concentrations of H_2_O_2_. The hydrogel was prepared by adding dropwise sodium alginate solution with rhodamine-bovine serum albumin to the CaCl_2_ solution to obtain millimeter-sized beads. Then, the beads were incubated in low molecular weight poly(vinyl alcohol) for 60 min. Finally, the beads were incubated with 1,3-benzenediboronic acid for 30 min. Another example of ionic crosslinking is a NIR-responsive hydrogel based on quaternized chitosan, tannic acid, and ferric ion (Fe(III)), which was developed by Guo et al. [29]. While the main backbone of the hydrogel consists of ionic bonds and hydrogen bonds between quaternized chitosan and tannic acid, metal coordination bonds between Fe(III) and catechol from tannic acid gave the hydrogel its adhesive and photothermal properties. The preparation of the hydrogel was conducted by mixing quaternized chitosan and FeCl_3_·6H_2_O, dissolving them in water, and then adding tannic acid solution to the mixtures.

Hydrophobic associations and van der Waals forces are other types of non-covalent bonds that are important for the stability of hydrogel networks and structures. Although these forces are weak, combining them with physical or chemical crosslinking mechanisms changes the dynamic behavior of smart hydrogels. Zhang et al. modified agar with three different anhydrides (maleic anhydride, succinic anhydride and glutaric anhydride) [30]. In the study, different concentrations of anhydrides were added to agar solution (10%, *w*/*v*) at 40 °C, following the addition of sodium hydroxide solution, while the pH of the reaction was maintained at 8–8.5. The study investigated the release of the hydrophobic active ingredient curcumin. Therefore, the hydrophobic structure of esterified agar promoted curcumin encapsulation, while the gel’s curcumin release is linked with pH changes.

**Figure 2:**
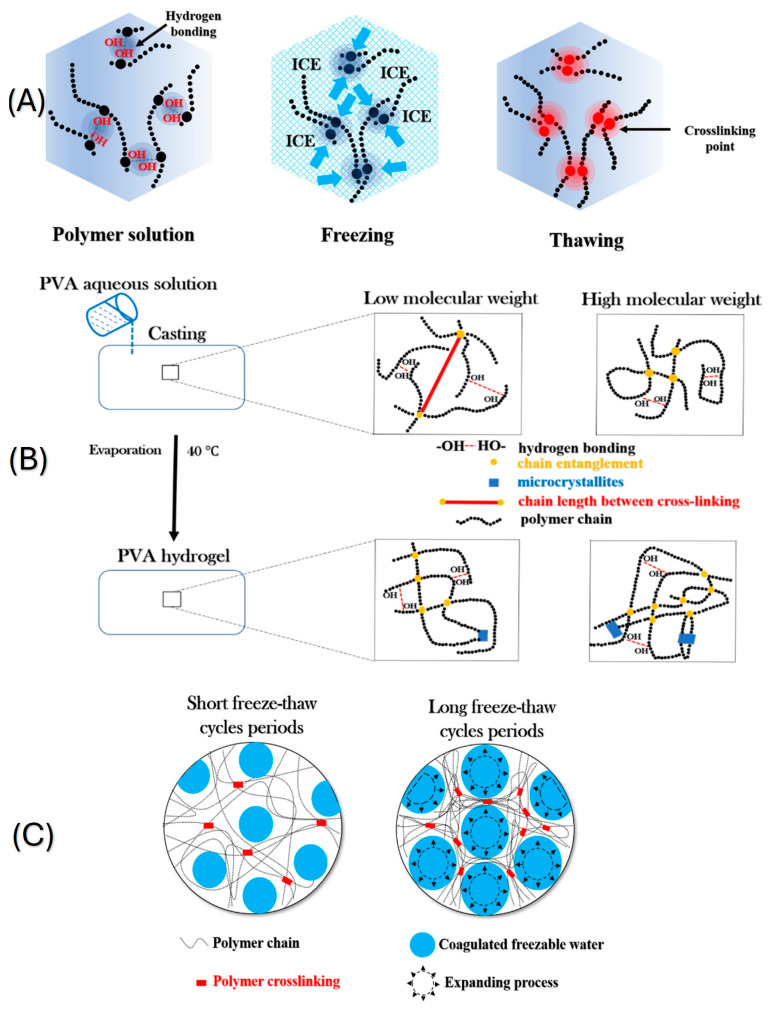
Freeze–thaw crosslinking and different effects on network formations of hydrogels, (**A**) Mechanism that shows how freezing and thawing contribute to crosslinking, (**B**) Effect of molecular weight on crosslinked polymer chains by freeze–thaw process, and (**C**) Effect of number of freeze–thaw cycles on crosslinked hydrogel network [33].

Freeze–thaw cycles were utilized to prepare hydrogels without the use of crosslinking agents, making the process straightforward. The mechanism of crosslinking with the freeze–thaw process is based on the formation and arrangement of microcrystals as shown in Figure 2. A hydrogel with tunable mechanical properties was prepared with freeze–thaw and solvent-exchange strategy [31]. In the study, firstly, curdlan was used to prepare organogels. Curdlan was dissolved in DMSO by stirring at 90 °C for 2 h. The organogels were degassed under vacuum conditions and then frozen at −20 °C and thawed at 25 °C. The prepared organogels were immersed in deionized water for 3 days, and the DMSO was replaced with fresh water three times each day at room temperature. Zhang et al. fabricated double-sensitive hydrogel by using oxidized cellulose nanofibers, polyvinyl alcohol and polydopamine coating [32]. 2,2,6,6-Tetramethyl-1-piperidinyloxy was used to oxidize microcrystalline cellulose to increase the pH sensitivity. pH and temperature-sensitive hydrogels were prepared by adding oxidized cellulose to poly(vinyl alcohol) water solution, and then the solution was subjected to five freeze–thaw cycles.

### 2.2. Chemical Crosslinking Methods

The preparation of smart hydrogels using chemical crosslinking methods typically involves the formation of covalent bonds. These methods usually involve a crosslinking agent. Due to the covalent bonds being stronger than that of physical interactions, hydrogels prepared with chemical crosslinking methods have higher mechanical strength. Additionally, utilizing chemical methods provides flexible strategies to adjust the properties and responsiveness of smart hydrogels easily. However, the usage of toxic chemical agents in the preparation of smart hydrogels may be a limiting factor for biomedical applications.

**Figure 3:**
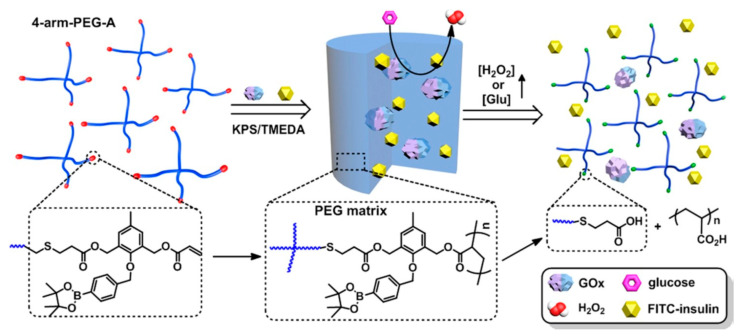
Redox-initiated radical polymerization for chemical crosslinking of 4-arm-PEG to prepare pH-sensitive smart hydrogel for insulin release [36].

Free radical polymerization is a process used to prepare hydrogels by chemically crosslinking. It is a method that provides robustness and affordability in the polymer industry [34]. N-isopropyl acrylamide-based pH and temperature-sensitive smart hydrogels were prepared by Isıkver et al. [35]. The hydrogels were prepared by dissolving N-isopropyl acrylamide, acrylamide, N,N′-methylenebisacrylamide and different dienoic acid co-monomer (acrylic acid or maleic acid), followed by adding ammonium persulfate and N,N,N′,N′-tetramethylethylenediamine. While N,N′-methylenebisacrylamide was used as a crosslinker, the free radical polymerization was carried out in a water bath at 70 °C. As shown in Figure 3, Zhang et al. prepared reactive oxygen species-responsive hydrogels through redox-initiated radical polymerization using 4-arm-poly(ethylene glycol) [36]. Before the preparation of H_2_O_2_-responsive hydrogel, 4-arm-PEG-SH was modified with H_2_O_2_-breakable acrylate and 1,3-diacryloxymethylbenzene, which is H_2_O_2_-insensitive. Hydrogels were prepared by using potassium persulfate to initiate the polymerization. Reaction was conducted at 4 °C for 48 h. Another example of free radical polymerization is the fabrication of electroactive hydrogels by UrRehman et al. [37]. Sodium dodecyl sulphate and NaCl were mixed in deionized water to form a surfactant solution. Lauryl methacrylate was added, followed by the addition of acrylamide to the surfactant solution. As a thermal initiator, ammonium persulfate was added. Arabic gum-stabilized multiwall carbon nanotubes were homogenized and added to the above-mentioned solution. Mixtures were put in plastic molds and kept at 60 °C for 30 min.

In contrast to the radical polymerization method, click chemistry is a process that involves simple reactions with high yields, producing selective products and eliminating toxic byproducts. Even though some materials (such as polysaccharides) are hard to apply click chemistry to, these polymers can be modified for the click reaction to be carried out. For the purpose of preparing a pH-sensitive hydrogel, Das et al. have implemented click chemistry on alginate and dextrin [38]. Dextrin was modified by the acetoacetylation process to obtain ketone-functionalized dextrin. Additionally, a hydrazide derivative of alginate was prepared as a gel precursor in the study. The ketone-functionalized dextrin and alginate hydrazide were simply mixed and stirred for 1 min to obtain the hydrogels. Another click chemistry-based pH-responsive hydrogel preparation was conducted by Ding et al. [39]. Firstly, C_6_ O-allyl chitosan, a pH-sensitive, UV-crosslinkable material, was synthesized by protecting the -NH_2_ groups and then undergoing a ring-opening reaction between the epoxy groups and the C_6_-OH. Gelation was performed by dissolving modified chitosan and exposing it to UV radiation (200 W) for 30 s.

The Michael addition reaction, which is the most common method for preparing sensitive hydrogel [40], was utilized by Liu et al. for the fabrication of a reduction-sensitive hydrogel [41]. Hyperbranched polyphosphoramidate and disulfide-modified hyaluronan were the components of the hydrogel. Thiol-acrylate hydrogels in the study were prepared by mixing a PBS solution of polyphosphoramidate and disulfide-hyaluronan, followed by adjusting the pH of the solution to 7.4 with 0.1 M NaOH. The hydrogels were obtained in air while stirring until the fluidity of the hydrogels could no longer be observed. Surfactant-free emulsion polymerization is another technique employed by Wei et al. to prepare temperature-responsive microgels [42]. N-Isopropylacrylamide and N,N′-methylenebisacrylamide were dissolved in deionized water in a two-neck reactor equipped with a reflux condenser and inlet-outlet for N_2_ bubbling. After mixing for 4 h at 70 °C, crosslinking was started by the addition of potassium persulfate to obtain microgels with a dense core. In the study, microgels with homogeneous crosslink distribution and poly(N-isopropylacrylamide)-co-poly(methacrylic acid) were also synthesized. Photopolymerization was another method for obtaining chemically crosslinked smart hydrogels. Schiphorst et al. fabricated light-responsive hydrogels through the copolymerization of spiropyran chromophores [43]. For the preparation of hydrogels, N-isopropylacrylamide, acrylic acid, N,N′-methylenebis(acrylamide), Irgacure 819 and synthesized spiropyran were dissolved in 2:1 dioxane-water. Another example of photocrosslinking is 5-fluorouracil-loaded pH-sensitive hydrogel, prepared by Aycan et al. [44]. While one of the components of the hydrogel, gelatin microspheres, was prepared using a water-in-oil emulsification process, the other component, methacrylated alginate, was synthesized by epoxide ring-opening reaction. The drug-loaded hydrogel system was fabricated through photocrosslinking using the photoinitiator Irgacure I2959 and under UV irradiation at 365 nm for 30 min. The carbodiimide crosslinking method is another method for preparing smart hydrogels. A glucose-responsive phenylboronic acid-based hydrogel was fabricated by Lu et al. [45]. ε-Polylysine and phenylboronic acid were crosslinked by 1-ethyl-3-(3-dimethylaminopropyl) carbodiimide and N-hydroxysuccinimide. 3-Fluoro-4-carboxy-phenylboronic acid and crosslinker agents were dissolved in DMSO and stirred at 20 °C for 1 h. On the other hand, polylysine was dissolved in deionized water and stirred under the same conditions. The first solution was added to the second solution slowly and mixed at 20 °C for 1 day. Afterwards, the mixture was dialyzed for 3 days and lyophilized. Hydrogel was prepared by mixing aqueous polylysine-phenylboronic acid solution with guar gum solution and stirring for 1 h.

The selection of the preparation method should be based not only on synthetic parameters but also on factors that determine antimicrobial performance. Physical crosslinking governs injectability, self-healing, adhesiveness, and dynamic porosity properties. These properties modulate drug retention and on-demand release in response to infection-relevant stimuli (pH shifts, ROS, enzymes, NIR). In contrast, chemical strategies enable the creation of precise network architectures with greater mechanical robustness and programmable degradation (diffusion- or erosion-controlled release). In this context, design strategies directly translate into clinically relevant functions, including stable yet responsive matrices for localized antibiotic delivery, photo-activated bacterial eradication (through photothermal/photodynamic mechanisms), anti-biofilm action, and tissue-integrative wound healing. This structure–process–function axis frames the analysis in the following sections, where antimicrobial smart hydrogels are examined according to their dominant polymeric platforms—alginate and chitosan—and fabrication choices are linked to their efficacy against Gram-positive/Gram-negative bacteria and fungi, as well as to outcomes in wound healing and tissue regeneration.

## 3. Types of Smart Hydrogels

Although four fundamental forces—ionic interactions, hydrophobic associations, hydrogen bonds, and van der Waals interactions—underlie hydrogel responsiveness, smart hydrogels are commonly classified into three main stimulus categories: physical (temperature, light, electric/magnetic fields, mechanical pressure/ultrasound), chemical (pH, ionic strength, chemical species), and biological (glucose, enzymes, antigens) stimuli as shown in Table 1 [46,47,48].

Thermoresponsive hydrogels represent the most extensively studied class of hydrogel systems. Their temperature-dependent behavior arises from thermally driven changes in polymer–water interactions, specifically temperature-dependent variations in hydrogen bonding and hydrophobic interactions, which determine whether water is retained within or expelled from the gel network. At low temperatures, hydrogen bonds and hydrophilic interactions dominate and bind water, whereas at elevated temperatures, hydrophobic interactions prevail and drive water expulsion from the polymer network. Thermoresponsive hydrogels undergo sol–gel phase transitions or volumetric swelling/deswelling above or below a characteristic critical solution temperature (CST) [49]. These hydrogels exhibit nonlinear, temperature-dependent responses and are categorized into two types based on their swelling principles: LCST-type and UCST-type hydrogels [50].

Lower Critical Solution Temperature LCST hydrogels exhibit volume collapse (phase transition) when the ambient temperature rises above the LCST because polymer solubility decreases. When the temperature exceeds LCST, hydrophobic interactions among the polymer chains’ apolar segments and hydrogen bonds with water weaken, leading to expulsion of absorbed water, gel collapse, and network contraction. Poly(N-isopropylacrylamide) (PNIPAM) is widely studied because its LCST (~32 °C) lies close to human body temperature (37 °C). Other examples include poly(vinyl methyl ether) (PVME). Most thermoresponsive hydrogels for biomedical applications are designed based on the LCST principle rather than the UCST principle, because many UCST systems have phase transition temperatures typically below 25 °C, which limits their biomedical utility. Thermoresponsive hydrogels can display sol–gel transitions that enable minimally invasive administration as liquids at low temperatures with gelation at body temperature. Network density, crosslink type, and the chemical structure of polymer chains tune LCST, mechanical strength, and biodegradability.

Upper Critical Solution Temperature UCST hydrogels show the opposite temperature dependence: increasing temperature increases polymer matrix solubility and the system undergoes a gel–sol transition at the UCST. These hydrogels undergo phase transition and collapse when cooled below the UCST. Examples of UCST-type hydrogels include polar zwitterionic poly(pentafluorophenyl acrylate), gelatin, and poly(acrylic acid) (PAA) hydrogels [51]. Copolymers of Nacryloylglycinamide (NAGA) and -Nacetylacrylamide (NAcAAm) also display UCST behavior. Interpenetrating polymer networks (IPNs) containing poly(acrylamide) (PAAm) and poly(acrylic acid) (PAAc-) have been reported to exhibit UCST near 25 °C [52].

Light is a promising stimulus due to its rapid response time, capability for remote actuation, suitability for miniaturization, tunable intensity, and the absence of a requirement for physical contact [53]. Light-responsive hydrogels that contain photoreactive moieties or photocleavable crosslinks exhibit rapid, remotely controlled responses upon irradiation at specific wavelengths [54]. These hydrogels are prepared by incorporating photoresponsive chromophores into the network structure [47]. They respond to external optical stimuli such as ultraviolet (UV) or visible light, and light-induced actuation can be applied instantaneously and transmitted with high spatial and temporal precision compared with stimuli limited by thermal or ionic diffusion [55]. Photochromic groups, such as azobenzenes, undergo reversible cis–trans isomerization under UV illumination, producing macroscopic volumetric changes in the hydrogel network [56]. Photocleavable functionalities, such as leuco-triarylmethane derivatives, can be incorporated into the network. Under UV irradiation, these groups photolyze into ion pairs, increasing osmotic pressure and driving swelling [50]. Photothermal effects are observed when light-absorbing chromophores (e.g., copper chlorophyllin trisodium salt) are embedded in thermoresponsive networks such as PNIPAM. Absorbed light (for example, 488 nm) is converted to heat locally, raising the hydrogel temperature and triggering the collapse of the thermoresponsive polymer matrix [53]. Photothermal agents (PTAs) incorporated in photothermal hydrogels can convert near-infrared (NIR) radiation into heat, enabling remote control of swelling/deswelling [54,57]. Supramolecular light-responsive systems exploit reversible host–guest interactions; for example, hydrogels formed from α-cyclodextrin and photoactive azobenzene derivatives show macroscopic, reversible deformation under 365 nm UV or 430 nm visible light. NIR-responsive compositions have been developed to enhance tissue penetration, such as PNIPAM nanocomposites containing graphene oxide (GO) nanoparticles that absorb NIR light and convert it to heat, enabling remotely controlled volume transitions. Incorporation of photocleavable moieties into the polymer network produces light-triggered ion pair formation and consequent osmotic swelling and electrostatic repulsion [46]. Metallic nanoparticles (Au, Pt, Ag, Cu) have also been used in the synthesis of light-sensitive hydrogels because of their strong light-absorption and scattering properties [58,59,60].

Electroresponsive hydrogels, also termed electro-sensitive hydrogels, are typically composed of polyelectrolytic polymers that contain polar or ionizable functional groups and undergo electrically driven volume, shape, or bending changes, thereby converting electrical energy into mechanical work [57]. Under an applied external electric field, mobile H^+^ and OH^−^ ions associated with the polymer network migrate toward oppositely charged regions, producing an asymmetric ion distribution. This ion redistribution increases the local osmotic pressure within the polymer matrix and induces macroscopic volume transitions, such as swelling or deswelling [61]. The electromechanical response is governed by the combined balance of forces, including osmotic pressure generated by ion gradients, polymer–polymer affinity, ionic pressure, rubber elasticity, and any electrochemical reactions at the electrodes. Electrically triggered actuation can produce rapid and pronounced volumetric changes; for example, sodium polyacrylate microcapsules have been reported to exhibit up to 96% volumetric change within 50 s under a DC density of 0.3 mA cm^−2^, driven by osmotic pressure differences arising from asymmetric ion distribution [57]. Composite formulations further expand functionality: polydimethylsiloxane (PDMS) hydrogels containing TiO_2_ particles demonstrate rapid and reversible bending toward the cathode under an applied electric field, with the bending direction reversible by alternating the electrode polarity [62]. Electroresponsive formulations can also modulate controlled drug release. Hydrogels based on chitosan-grafted polyaniline copolymers and oxidized dextran show voltage-dependent release profiles, with an increasing applied potential significantly enhancing the release of model drugs such as amoxicillin and ibuprofen [63].

Magnetic field-responsive hydrogels (magneto-responsive hydrogels) are typically fabricated by incorporating magnetic nanoparticles into a thermoresponsive hydrogel matrix [57]. For example, embedding superparamagnetic iron oxide nanoparticles (SPIONs) within a PNIPAAm thermoresponsive network yields a composite hydrogel that responds to an applied magnetic field. Under alternating magnetic fields, SPIONs oscillate or relax, generating localized heating (magnetic hyperthermia). The heat activates the thermoresponsive component, altering the swelling behavior and triggering gel–sol or sol–gel transitions, thereby modulating the release rate of incorporated therapeutic agents [46,50,63].

Mechanical strain, pressure, or ultrasound can be used to trigger hydrogel systems for controlled, scheduled, or activity-dependent drug release. Elastic films or microcapsules loaded with nanoparticles function as mechanically responsive carriers. Application of tensile or shear stress increases the exposed surface area of an elastic, elastomeric, or hydrogel matrix, thereby enhancing diffusion and initiating payload release. In strain-responsive constructs, drug-loaded nanoparticles can be embedded within microaggregates or microdeposits. Mechanical loading causes the rupture or fragmentation of these aggregates, producing a mechanically gated, dose-controlled release mechanism that can be targeted to high-shear vascular regions. Ultrasound exposure increases hydrogel permeability by imparting mechanical stress, localized heating, and cavitation, thereby enabling spatially and temporally targeted release. Ultrasound-mediated effects include transient pore formation, accelerated diffusion, and mechanical disruption of encapsulating structures, all of which facilitate on-demand drug liberation. Representative studies demonstrate that ultrasound application to insulin-loaded hydrogels based on HEMA copolymers results in reproducible and controlled insulin release under clinically relevant insonation conditions [49,64,65].

pH-responsive hydrogels are among the most extensively investigated classes of smart hydrogels for controlled and targeted drug delivery [57]. Their polymer networks incorporate weakly acidic functional groups (for example, carboxylic or sulfonic acids) or weakly basic functional groups (for example, amines) whose ionization state changes with the ambient pH. The degree of ionization of these moieties depends on the environmental pH relative to the polymer’s pKa or pKb. Anionic hydrogels possess polyacidic character. When the environmental pH rises above the polymer pKa, carboxyl groups deprotonate, increasing interchain electrostatic repulsion and osmotic pressure and producing pronounced swelling. Cationic hydrogels possess polybasic character. When the environmental pH falls below the protonation threshold of the basic groups, these moieties become protonated, generating a positive charge and driving the swelling process. Because protonation–deprotonation processes are reversible, cyclic pH changes can repeatedly and predictably modulate the swelling and deswelling behavior of the material. For example, in poly(acrylic acid) and related ionizable polymers, increasing the solvent pH above the polymer pKa causes deprotonation of carboxyl groups and accumulation of negative charge; the resulting increase in charge density and osmotic pressure promotes extensive water uptake and hydrogel swelling, whereas acidification reprotonates the groups and induces network contraction. The reversibility of the protonation–deprotonation equilibrium enables facile and repeatable pH-driven volume transitions. Advanced formulations utilize pH-sensitive chemistries to achieve ultra-responsive behavior and additional functionalities. Aldehyde-functional polymers and amine-modified silica nanoparticles have been employed to create injectable, self-healing hydrogels that display rapid pH-triggered responses, making them suitable for biomedical delivery and tissue engineering applications [47,51,57,61,66].

Ionic strength-responsive hydrogels are polymer networks containing ionizable functional groups that undergo reversible swelling or deswelling in response to changes in the ionic concentration of the surrounding solution. Variations in ionic strength modulate electrostatic interactions and the osmotic pressure within the network, thereby directly altering the hydrogel’s water-holding capacity. Elevated concentrations of dissolved ions partially neutralize or screen the charges on polymer side groups, reducing interchain electrostatic repulsion and limiting swelling through a charge-masking effect. Multivalent counterions can act as physical crosslinkers, promoting more pronounced network collapse. Although non-ionic polymer matrices generally exhibit weaker sensitivity to salt concentration, specific systems display sharp volume phase transitions at critical ion concentrations. Ionizable polymer chemistries commonly used for ionic strength responsiveness include acrylic acid derivatives and composite architectures, such as NIPAM/AA bilayer hydrogels, which can produce reversible folding and unfolding motions in response to changes in ionic strength. Formulations incorporating polysaccharides or reactive additives (for example, xanthan gum coupled with succinic anhydride derivatives) have been investigated for ion force-sensitive, controlled release applications [50].

Redox-responsive hydrogels respond to variations in the local cellular redox environment. They incorporate redox-labile linkages such as disulfide bonds or metal-ligand coordination motifs that are cleaved or reorganized under reducing conditions, resulting in network degradation or alteration of mechanical properties. For example, polyethylene glycol (PEG) hydrogels crosslinked by disulfide bridges, which are reducible by intracellular glutathione (GSH), have been developed for tissue regeneration and targeted drug delivery applications. Redox cycling of transition metal ions, such as the Fe^3+^/Fe^2+^ couple, can also be exploited to impart tunable mechanical properties and redox-sensitive behavior to hydrogel matrices [49,51,65,67,68].

Biological triggers are specific molecules or conditions in living systems, including glucose, enzymes, antigens, nucleic acids, and redox agents. Hydrogels responsive to biological cues modulate their structural integrity, swelling/deswelling behavior, or ligand-induced conformational states in response to these signals, thereby adjusting their mechanical properties and the release profile of encapsulated payloads. Enzyme-sensitive hydrogels are engineered to respond to the presence of defined proteases such as matrix metalloproteinases (MMPs). Enzyme-cleavable peptide sequences are incorporated as crosslinkers within the network; enzymatic hydrolysis of these motifs leads to network degradation, increased porosity, payload release, and mechanical softening. Such systems are particularly suited to tumor microenvironment-responsive drug delivery, regenerative scaffolds that remodel with cell infiltration, and anti-adhesion coatings. Glucose-responsive hydrogels aim to provide autonomous control of insulin delivery for the management of diabetes. Typical strategies employ phenylboronic acid (PBA) moieties that reversibly form diol complexes or incorporate glucose oxidase (GOx) enzymes, which convert glucose to gluconic acid, thereby altering the local pH. Glucose-induced PBA complexation or enzyme-mediated reactions change network permeability, swelling degree, or carrier release kinetics. Representative formulations include pH-sensitive peptide hydrogels loaded with insulin and GOx/catalase enzyme systems, as well as chitosan/PVA hybrids functionalized with PBA for glucose-controlled release. Antigen-responsive hydrogels utilize specific antigen–antibody or aptamer–target interactions to produce selective and reversible changes in hydrogel volume or permeability in the presence of the target molecule, enabling target-triggered release or capture. DNA-based hydrogels leverage single or double-stranded oligonucleotide sequences conjugated to the polymer network; sequence-specific hybridization events reversibly modulate swelling, collapse, or phase transitions. Aptamer-conjugated hydrogels have been applied for toxin detection, targeted cell capture, and controlled release in response to specific molecular targets [50,54,55,69,70].

Table 2 summarizes the advantages and principal challenges or limitations (disadvantages) associated with the principal smart hydrogel types reported in the literature, organized by stimulus modality:

## 4. Applications of Smart Hydrogels for Microbial Diseases

The effectiveness of hydrogels depends not only on their ability to respond to the microenvironment but also on the nature of the pathogens involved. Understanding the structure and composition of microbial membranes is crucial for designing more selective and effective hydrogels. In the case of Gram-positive bacteria, such as *Staphylococcus aureus* and *Staphylococcus epidermidis*, the cell wall is composed of a thick layer of peptidoglycan along with teichoic acids and lipoteichoic acids, which confer mechanical resistance and adhesive capacity [75]. However, this negatively charged structure also makes them vulnerable to cationic polymers, such as chitosan, which can interact with their membranes and compromise their viability.

In contrast, Gram-negative bacteria, such as *Escherichia coli* and *Pseudomonas aeruginosa*, possess a cell envelope composed of an outer membrane located over a thin layer of peptidoglycan. Intercalated between the outer membrane and the plasma membrane lies the periplasmic space, which constitutes an integral compartment of the cell wall and plays a critical role in the cell’s energy metabolism. For this reason, Gram-negative bacteria exhibit a greater intrinsic resistance to many antibiotics [75]. Nevertheless, the abundance of negatively charged lipopolysaccharides (LPS) in the outer membrane facilitates interaction with positively charged polymers, thereby enhancing the antimicrobial effect of certain hydrogels.

Finally, fungi of the genus Candida, such as *C. albicans*, *C. krusei*, and *C. glabrata*, possess a cell wall that functions as a protective barrier, limiting solute access to the plasma membrane. This wall is composed of a layer of chitin, whose proportion varies among fungal species and is directly associated with the cell membrane. Beyond this layer lies a network of β-glucans, linked together by β-(1 → 6) bonds, which account for approximately 50–60% of the cell wall. On the extracellular side of the wall, glycoproteins are found, representing between 20% and 60% of the total wall composition [76,77,78]. Although this structure renders *Candida* species resistant to many antibacterial agents, it also opens the possibility of designing hydrogels capable of altering wall permeability or interfering with biofilm formation, one of the main virulence strategies of these yeasts.

This biological framework explains why some polymers display greater antimicrobial efficacy than others. The choice of material determines not only biocompatibility and responsiveness to specific stimuli, but also the efficiency of controlled drug release and the interaction with the infectious microenvironment. Among natural polysaccharides, alginate, chitosan, hyaluronic acid, and cellulose stand out for their availability, low cost, and versatility in biomedical applications.

This review focuses specifically on alginate- and chitosan-based stimuli-responsive hydrogels, as these two polymers represent the most extensively studied and functionally complementary materials in the development of hydrogel systems with antimicrobial activity, as highlighted in several recent reviews on smart and biopolymer-based hydrogels (REF). Additionally, a bibliometric analysis was carried out on the Scopus database, which can be seen in Table 3 covering publications between 2017 and 2025 related to “stimuli-responsive antibacterial hydrogels.” The results showed that 41% of these publications correspond to alginate-related systems and 65% to chitosan-related systems, highlighting the significance of these polymers in this field of research.

In most cases, both materials act as primary polymers within hybrid formulations, being combined with other natural or synthetic polymers, organic compounds, or inorganic nanomaterials to enhance specific properties such as mechanical stability, controlled release, or antimicrobial activity. Alginate is an anionic, biocompatible polysaccharide with adjustable ionic crosslinking capacity. It also exhibits high drug-loading efficiency, making it an ideal material for controlled and sustained drug delivery systems. Conversely, chitosan is a cationic polymer with intrinsic antimicrobial properties and bioadhesive behavior derived from its protonated amino groups, which interact with negatively charged bacterial surfaces.

These complementary properties make alginate and chitosan ideal reference models for understanding how polymer composition, charge distribution, and structural modifications influence antimicrobial activity and therapeutic outcomes. Furthermore, both polymers can be combined with other systems, enabling the incorporation of advanced functionalities such as tissue regeneration capability, infection detection, or dynamic responsiveness to physiological stimuli, thereby broadening their potential in biomedical applications. Therefore, structuring the discussion around these two representative systems allows for an integrated comparison of design strategies, mechanisms of action, and clinical performance across different infection models. Although other natural or synthetic polymers (e.g., hyaluronic acid, cellulose, or PLGA) have been explored, their use is generally as complementary components rather than main structural matrices, reinforcing the dominant role of alginate and chitosan in stimuli-responsive hydrogel systems.

### 4.1. Alginate-Based Hydrogels

Alginate is a natural polysaccharide primarily obtained from brown algae of the class *Phaeophyceae*, although it can also be produced by certain bacteria such as *Azotobacter vinelandii* and *Pseudomonas aeruginosa*. Structurally, it is composed of two monomers: mannuronic acid (M units) and guluronic acid (G units). These units are capable of interacting with divalent cations, particularly calcium (Ca^2+^), leading to the formation of three-dimensional matrices with a high-water retention capacity [79,80]. This property makes alginate gels suitable materials for biomedical applications, particularly in wound treatment. Moreover, their structure enables the stable incorporation of antibiotics and peptides, thereby enhancing their use as carriers in controlled drug delivery systems.

Despite these advantages, alginate-based three-dimensional networks often present mechanical and stability limitations. Alginate hydrogels tend to be fragile, with low tensile strength and a rapid degradation rate under physiological conditions, which may compromise their performance in long-term biomedical applications [81,82]. For this reason, alginate is frequently combined with other natural or synthetic polymers, such as chitosan, hyaluronic acid, or polyvinyl alcohol (PVA), which provide greater mechanical strength, elasticity, and improved control over drug release kinetics. In addition, these polymers enable the introduction of dynamic linkages or additional chemical functionalizations, thereby improving responsiveness to specific stimuli (pH, ROS, enzymes, light) [83,84,85,86,87]. Thus, reinforcement with complementary materials transforms alginate into a more robust and versatile platform for designing of smart hydrogels with advanced antimicrobial applications.

Table 4 summarizes the most relevant findings from the studies analyzed between 2017 and 2025, organized by type of formulation and the agents incorporated. In this way, it facilitates the comparison among different alginate-based hydrogel designs, highlighting how chemical modifications and the combination with other polymers directly influence their rheological properties, antimicrobial capacity, and reported clinical applications.

It is possible to delve deeper into the most representative contributions of each study, analyzing in greater detail the structural and functional aspects that determined the antimicrobial performance of the employed hydrogels. In this context, one of the first noteworthy works is that of Wan et al., in which a supramolecular hydrogel based on lysine-rich amphiphilic peptides (APs), reinforced with sodium alginate, was developed [88]. Hydrogel formation occurs in aqueous solution, where the APs self-assemble through hydrophobic interactions and hydrogen bonding between lysine chains, generating supramolecular fibers that organize into a three-dimensional network. This network exhibits pH sensitivity: under slightly acidic conditions, the protonation of the amino groups stabilizes the structure and enhances its antimicrobial activity. However, when the hydrogel is prepared using APs alone, the resulting structures are mechanically weak; therefore, alginate is incorporated as a structural reinforcement. Owing to its negatively charged carboxylate groups, alginate interacts electrostatically with the protonated amino groups of lysine polymers, strengthening the supramolecular network. This association improves the mechanical strength, viscosity, and rheological properties of the hydrogel, facilitating its handling and application. The hydrogels obtained demonstrated antibacterial activity against *Escherichia coli*, attributed to the interaction of free amino groups with the anionic bacterial membranes, leading to their destabilization and subsequent cell death. These findings confirm the potential of this hybrid system for biomedical applications in the treatment of infected wounds.

The skin is the largest organ of the body and one of the most important, as it is responsible for protecting and interacting with the organism’s external environment. When a laceration or wound occurs, whether due to accidental or surgical causes, the cutaneous barrier is compromised, thereby increasing the risk of infection by microorganisms, both exogenous and those belonging to the normal skin microbiome. Among the most prevalent opportunistic pathogens are *Candida albicans*, with 5142 cases of invasive candidiasis reported in the United Kingdom in 2016 [94], and *Staphylococcus aureus*, which causes severe skin infections in susceptible patients such as those with burns or under immunosuppression [95]. To prevent such infections, the use of wound dressings that promote the healing process is common in the treatment of skin injuries. Moist dressings have proven effective in accelerating wound closure, which explains the increasing research interest in hydrogels as wound dressings in recent years. Among these studies, the work of stands out, in which an injectable and antibacterial hydrogel was designed by incorporating aminoglycoside antibiotics into the polymeric network [89]. These antibiotics fulfilled a dual function: they were gradually released from the matrix while also acting as crosslinking agents, becoming structurally integrated into the hydrogel network. In this system, antibiotic release occurs primarily through the controlled erosion of the matrix, in contrast to classical diffusion mechanisms, which are slower and limited by pore size.

To enable the incorporation of antibiotics into the network, oxidized forms of polymers such as alginate, carboxymethylcellulose, chondroitin, and dextran were employed. These polymers contain aldehyde groups that react with the amino groups of aminoglycosides, generating Schiff bases responsible for the crosslinking of the hydrogel. The gelation time and the resulting mechanical properties depend on the number of amino groups in the antibiotic, the type of polymer used, and its degree of oxidation.

In this study, nine aminoglycosides with different numbers of amino groups were evaluated [89]. Neomycin, with six amino groups, exhibited the shortest gelation times, achieving hydrogel formation in approximately 20 s when crosslinked with oxidized dextran. In contrast, amikacin, which contains four amino groups, required about one minute to gel under the same conditions. The type of polymer also had a significant influence on the process: among the materials tested, oxidized alginate displayed the longest gelation times, which was most likely due to its lower hydrophobicity and higher viscosity, both factors that slow the interaction between functional groups. Furthermore, it was observed that a higher degree of oxidation (i.e., a greater number of available aldehyde groups) reduced the gelation time, thereby favoring network formation.

The antibacterial effect of these hydrogels is attributed to the cleavage of the bonds formed between the antibiotic and the polymer under the acidic microenvironment characteristic of infected tissues, which facilitates a targeted release of the antibiotic. In this way, the bacterial infection itself accelerates gel degradation and drug release; once the bacteria are eliminated and tissue pH is restored, the release process slows down. Antibacterial activity was evaluated by measuring the percentage of bacterial survival. As model hydrogels, those obtained with amikacin were employed, and the bacterial strains analyzed included *Escherichia coli*, *Staphylococcus aureus* (USA300 strain), *Staphylococcus epidermidis*, and *Pseudomonas aeruginosa* (PAO1). The results showed that bacterial survival was below 3% in the presence of these hydrogels. Moreover, in vivo assays performed in female Kunming mice with skin wounds infected with *S. aureus* confirmed the treatment’s efficacy, demonstrating the ability of these systems to control infections and promote wound healing.

Another study focused on the design of hydrogels for the treatment of skin wounds and infections is that of Hu et al., in which an injectable hydrogel was developed from alginate modified with phenylboronic acid groups (ALG-BA) [96]. These groups are capable of establishing dynamic boronate-ester linkages that are sensitive to pH and reactive oxygen species (ROS). As a multiple-loading strategy, amikacin was directly incorporated into the polymeric matrix, while naproxen was encapsulated in hyaluronic acid-cholesterol (HA-CH) micelles, which were subsequently integrated into the hydrogel. The resulting system exhibited rapid gelation, self-healing capacity, and injectability, positioning it as a versatile platform for biomedical applications.

The physicochemical characterization of this hydrogel showed that it possessed mechanical stability and enabled the controlled release of drugs. This release was modulated by microenvironments with acidic conditions (typical of bacterial metabolism) and elevated ROS levels, which accelerated the release of both amikacin and naproxen. In in vitro assays, the hydrogel demonstrated potent antibacterial activity, achieving 90% inhibition against Staphylococcus aureus and 98% inhibition against Pseudomonas aeruginosa. In vivo studies, using an infected wound model in rats, confirmed these findings, showing accelerated wound healing accompanied by a significant reduction in inflammatory mediators (TNF-α) and an increase in IL-10, reflecting a synergistic effect of antimicrobial control and modulation of the inflammatory response.

On the other hand, in the study by Mai et al., a hybrid hydrogel was designed consisting of carboxymethyl chitosan (CMCS) and alginate, specifically oriented toward the treatment of skin wounds caused by burns [91]. In its formulation, the photosensitizer sinoporphyrin (DVDMS) was incorporated for photodynamic antimicrobial chemotherapy (PACT), along with poly(lactic-co-glycolic acid) (PLGA) nanoparticles loaded with basic fibroblast growth factor (bFGF). Preparation was carried out through the controlled mixing of the biopolymers and the encapsulation of DVDMS and bFGF, resulting in a stable and biocompatible three-dimensional network. Characterization studies confirmed that the hydrogel exhibited excellent swelling capacity, adequate mechanical strength, and sustained release of both the photosensitizer and the growth factor, establishing it as a multifunctional platform for antimicrobial and tissue regeneration applications in skin.

The hydrogel exhibited stable behavior under physiological conditions and enabled the controlled release of DVDMS and bFGF, which was essential to combine antimicrobial action with the promotion of wound healing. DVDMS is a compound that acts as a photosensitizer for photodynamic antimicrobial chemotherapy (PACT); when activated by low-intensity light, it generates reactive oxygen species (ROS) capable of damaging bacterial membranes and cellular structures. This strategy resulted in potent antimicrobial activity, achieving in vitro eradication of 99% of multidrug-resistant Staphylococcus aureus (MDR-*S. aureus*). In in vivo assays using an infected burn model, the treatment not only effectively eliminated the bacterial infection due to the action of DVDMS but also promoted angiogenesis, cell proliferation, and accelerated tissue repair through the effect of bFGF. Taken together, the system demonstrated a combined and multifunctional strategy, capable of integrating photodynamic antibacterial activity with the stimulation of tissue regeneration in skin wounds.

In a more recent study, Dong et al. developed a multifunctional hydrogel based on alginate and poly(2-(methacryloyloxy)-N,N,N-trimethylethanaminium chloride) (PDMC), a quaternary cationic polymer, reinforced with polydopamine (PDA) [92]. The system was designed for the treatment of infected skin wounds, simultaneously integrating antimicrobial, photothermal, and sensing properties. The hydrogel network was formed through ionic crosslinking, with PDA acting as a key component to provide adhesive capacity, photothermal effect under near-infrared (NIR) irradiation, and electrical conductivity. The resulting hydrogel was flexible, self-healing, and able to adapt to the wound bed, while also maintaining a high water retention capacity, a critical condition for wound healing. Furthermore, the hydrogel exhibited mechanical resistance and photothermal properties; upon NIR irradiation, PDA absorbed energy and generated localized heat, thereby enhancing the bactericidal effect of the system. In in vitro assays, inhibition of *Staphylococcus aureus* and *Escherichia coli* growth was observed. In vivo studies, using an infected wound model in rats, showed accelerated and nearly complete wound closure (approximately 96–100% within 14 days), along with a reduction in bacterial load. Additionally, the hydrogel demonstrated the ability to function as a sensor for pressure, strain, and temperature.

In the work of Al-Zuhairy et al., a hydrogel composed of poly(lactic-co-glycolic acid) (PLGA)/alginate/reduced graphene oxide (r-GO) was designed, incorporated into a neem oil emulsion, to develop a therapy targeted at mucosal infections, specifically otitis media [93]. The system included hesperidin, a flavonoid with antioxidant and anti-inflammatory properties, encapsulated within the GO nanocarrier and coated with the PLGA-alginate matrix. The hydrogel network was formulated to be pH-responsive, thereby accelerating the release of active compounds under acidic conditions typical of infected sites. Characterization studies showed that the hydrogel maintained structural stability, swelling capacity, and sustained release of hesperidin (82% at pH 5.4 vs. 65% at pH 7.4 over 96 h). The system exhibited multifunctional activity: beyond controlled release, it provided antioxidant and anti-inflammatory properties. In in vitro assays, activity against *Staphylococcus epidermidis* and *Candida albicans* was demonstrated, with significant inhibition of biofilm formation. In vivo assays, using a rat model of otitis media, confirmed therapeutic efficacy, with a reduction in pro-inflammatory mediators (IL-1β, TNF-α, TLR4) and an increase in antioxidant factors such as Nrf-2 and SOD.

### 4.2. Chitosan-Based Hydrogels

Chitosan is a polysaccharide derived from the deacetylation of chitin, characterized by its biocompatibility and its ability to form three-dimensional networks. Unlike other biopolymers, it possesses an intrinsic antimicrobial activity attributed to its positively charged amino groups, which interact with bacterial membranes, leading to destabilization and cell death. Several studies have demonstrated its efficacy against a broad spectrum of microorganisms, including Gram-negative bacteria such as *E. coli* and Gram-positive bacteria such as *S. aureus* [97,98]. Likewise, antifungal activity has been reported for chitosan and its derivatives, such as carboxymethyl chitosan, against yeasts including *C. albicans*, *C. krusei*, and *C. glabrata* [99]. These properties have fostered a growing interest in the development of chitosan-based hydrogels, either in pure formulations or in combination with other polymers, with the aim of improving cell adhesion, hydration capacity, and overall performance in the treatment of infected wounds and in the controlled release of therapeutic agents [100].

The reviewed studies show that chitosan-based hydrogels are highly versatile in both structure and biomedical use. Early work by Qu J. et al. combined chitosan with conductive polymers and oxidized dextran to create injectable, biocompatible, and biodegradable hydrogels [101]. These hydrogels could release antibiotics like amoxicillin and ibuprofen in a controlled way, responding to changes in pH and electric field, and showed antibacterial effects against *S. aureus* and *E. coli*.

Subsequent studies, such as that of Wang et al., further explored the potential of smart hydrogels to integrate both diagnostic and therapeutic functions [102]. Their hybrid formulation of chitosan with bromothymol blue and conjugated polymers enabled in situ visual detection of infection and the eradication of bacterial biofilms through photothermal activity under NIR irradiation, showing efficacy against *S. aureus*, *K. pneumoniae*, and ampicillin-resistant *E. coli*. Similarly, Liang et al. proposed an adhesive hydrogel based on quaternate chitosan, protocatechualdehyde, and ferric ions, stabilized through dynamic Schiff base and catechol–Fe coordination bonds [103]. This system exhibited self-healing capacity, strong tissue adhesion, and controllable removability, together with potent antimicrobial activity against methicillin-resistant *S. aureus* (MRSA) and *E. coli*, confirmed in infected wound models in rats.

Recently, hydrogel designs have become more multifunctional and targeted. For example, Yan et al. [104] added mupirocin to a hydrogel made of polyacrylate, quaternized chitosan, and ZnO nanoparticles, resulting in stronger materials that release drugs selectively in inflamed areas and fight *S. aureus* and *E. coli*. Madivoli et al. [105] and Torabiardekani et al. [106] combined chitosan with cellulose, polyvinyl alcohol, gelatin, ZnO nanostructures, or essential oils to create hydrogels with antibacterial and antifungal effects, biocompatibility, and temperature-controlled drug release.

In 2024, Su et al. advanced toward the integration of theragnostic platforms, reporting a multilayer hydrogel combining chitosan, agarose, and phthalocyanine [107]. This system was capable of real-time infection detection through colorimetric changes, while simultaneously eradicating *S. aureus* biofilms via photodynamic therapy, achieving inhibition rates close to 99.9%.

The studies published in 2025 reflect the maturity and specialization of chitosan hydrogel applications. Emad et al. designed a dual pH- and temperature-responsive hydrogel loaded with naringenin and ferulic acid, which displayed antimicrobial activity against *B. subtilis* and *E. coli* [108]. In diabetic rat ulcer models, this system promoted re-epithelialization, angiogenesis, and reduced inflammation. Kamel et al. reported a chitosan–PVP hydrogel loaded with zinc-based MOFs and silver nanoparticles, exhibiting broad-spectrum antibacterial and antifungal activity (against *S. aureus*, *E. coli*, and *F. solani*) without cytotoxic effects while also supporting wound healing in animal models [23].

Other studies focused on specific clinical uses. Xiaojie et al. and Zhang et al. [109,110] developed a temperature-sensitive hydrogel with silver nanoparticles and antioxidants for the treatment of periodontitis. It worked against *P. gingivalis* and supported antibacterial, anti-inflammatory, and bone-regenerating effects. Zhang et al. created an injectable hydrogel for bone repair, utilizing carboxymethyl chitosan, silk fibroin, and bioactive glass [110]. This material fought *S. aureus* and *P. gingivalis* and promoted bone and blood vessel growth, like commercial collagen membranes. Fang et al. developed a smart hydrogel that responds to bacteria, composed of gelatin–chitosan with polydopamine and quaternate chitosan and PMAA [111]. This system was highly effective against MRSA, providing long-lasting protection, pH-triggered bacteria killing, and tissue repair in infected wounds.

Table 5 presents key studies from 2018 to 2025 on chitosan-based hydrogels for infection control. These studies demonstrate that researchers have employed a range of strategies, including chemical modifications, the addition of bioactive agents, and the creation of stimulus-responsive or multilayered designs. These methods have expanded the biomedical uses of chitosan. The selected studies encompass hydrogels for wound and skin infection dressings, as well as more specialized applications in periodontitis and bone regeneration, providing a comparison of their structures, antimicrobial effects, and therapeutic potential.

A comparative analysis of the studies presented in Table 4 and Table 5 reveals that the biological performance of stimuli-responsive hydrogels depends not only on their structural design but also on the morphological and physicochemical characteristics of the microorganisms evaluated.

Due to the anionic nature of alginate, which resembles the negatively charged surface of bacterial cell walls, alginate-based hydrogels alone are not expected to exhibit significant antimicrobial activity. However, they serve as highly efficient platforms for the controlled release of bioactive compounds, particularly when the objective is to simultaneously modulate antimicrobial and regenerative processes. The negatively charged alginate matrix enables the immobilization of cationic molecules such as antimicrobial peptides or aminoglycoside antibiotics, which can be integrated into the polymeric network and demonstrate efficacy against both Gram-positive (*S. aureus*) and Gram-negative (*E. coli*, *P. aeruginosa*) bacteria. In these systems, pH- or ROS-triggered release allows for site-specific activity within the infectious microenvironment, while formulations incorporating photosensitizers or polydopamine enhance the activity against resistant strains and biofilms through photodynamic or photothermal mechanisms.

In contrast, chitosan-based hydrogels possess intrinsic antimicrobial activity arising from their protonated amino groups, which establish electrostatic interactions with negatively charged bacterial membranes. This interaction leads to membrane disruption and loss of permeability, an effect particularly pronounced in Gram-positive bacteria, whose thick peptidoglycan and teichoic acid layers favor electrostatic attraction with cationic polymers. Conversely, the presence of an outer lipid membrane in Gram-negative species such as *P. aeruginosa* often restricts the diffusion of macromolecules, requiring smaller pore sizes or dual-mechanism systems—such as pH- or ROS-triggered release—to achieve effective antimicrobial action. Chemical modifications, such as chitosan quaternization, further enhance this affinity and extend the antimicrobial spectrum toward Gram-negative bacteria. At the same time, the incorporation of metallic nanoparticles (ZnO, AgNPs, MOFs) or photosensitizers broadens the efficacy against fungal pathogens (e.g., *Candida* spp.) and anaerobic bacteria such as *P. gingivalis*, which possess more complex envelopes or mature biofilms.

Overall, in both alginate- and chitosan-based hydrogels, design parameters (including crosslinking density, pore size, charge distribution, and the integration of dynamic bonds or bioactive compounds) determine the system’s capacity to selectively interact with different microbial types, overcome structural barriers such as the outer membrane of Gram-negative bacteria or the chitin layer of fungi, and maintain a controlled, stimulus-responsive antimicrobial effect under physiological conditions. This comparative perspective provides valuable guidance for the rational design of next-generation smart hydrogels, where fine-tuning of polymer structure and charge distribution can be tailored to target specific classes of pathogens while minimizing cytotoxicity and promoting tissue regeneration.

Based on the analysis of recent studies on smart hydrogels, it is evident that a thorough understanding of the structural and physicochemical characteristics of the polymers that form the matrix, as well as of the compounds combined with them, is crucial to enhancing the functionality of the resulting materials. These synergistic interactions enable the development of multifunctional hydrogels that integrate complementary properties such as tissue regeneration, modulation of the inflammatory response, and promotion of wound healing, while maintaining effective and controlled antimicrobial action.

One of the main causes of antimicrobial resistance (AMR) is the indiscriminate use of antibiotics in the treatment of infectious diseases. In this context, the development of hydrogel-based systems capable of achieving controlled drug release, maintaining therapeutic concentrations within an optimal range, and extending the interval between doses represents a significant step forward in AMR management. Moreover, employing these matrices as delivery platforms for new compounds that have demonstrated efficacy against multidrug-resistant strains may contribute to reducing the rate of resistance development.

In recent years, these systems have evolved from materials designed solely for the controlled release of bioactive compounds to more complex and interactive therapeutic platforms capable of dynamically responding to biological conditions. Nevertheless, important challenges remain regarding reproducibility, scalability, and in vivo validation, as well as the optimization of polymer architecture and charge distribution to specifically target multidrug-resistant pathogens without compromising biocompatibility. In this regard, the development of next-generation smart hydrogels that integrate therapeutic, diagnostic, and regenerative functions within a single platform represents a logical step toward clinically relevant systems for infection management and regenerative medicine.

## 5. Conclusions

The rising challenge of antimicrobial resistance (AMR) necessitates the development of innovative therapeutic strategies that go beyond conventional antibiotics. Smart hydrogels emerge as a powerful platform to address this challenge by enabling targeted, controlled, and multifunctional treatment modalities, thereby enhancing efficacy while minimizing systemic toxicity and the potential for further resistance development.

The efficacy of these advanced systems is fundamentally dictated by material selection and formulation. While natural polymers such as alginate and chitosan provide a biocompatible and versatile foundation, their inherent limitations are strategically overcome through chemical modification and compositing. Alginate-based hydrogels stand out for their versatility in controlled drug delivery, whereas chitosan-based systems exhibit intrinsic antimicrobial activity derived from their cationic nature. Recent advances have integrated these polymers with bioactive molecules, photosensitizers, or inorganic nanomaterials, generating hybrid matrices capable of on-demand release, biofilm eradication, and tissue regeneration—positioning them as next-generation therapeutic and diagnostic tools.

The future of antimicrobial hydrogels is oriented toward multifunctionality, personalization, and clinical translation. Beyond controlled release, the next generation of hydrogels is expected to integrate diagnostic, regenerative, and immunomodulatory functions into a single platform. Despite the abundant literature on smart hydrogels, their clinical application still faces major challenges such as reproducibility, scalability, regulatory approval, and long-term biocompatibility. Overcoming these barriers will require interdisciplinary collaboration among chemists, bioengineers, clinicians, and national and international regulatory agencies to ensure the safety, efficacy, and traceability of new biomedical systems.

Likewise, a deeper understanding of polymer–cell interactions, including their influence on cell adhesion, proliferation, and differentiation, will be crucial for the rational design of clinically effective hydrogels. A detailed understanding of the chemical structure, physicochemical properties, and biological interactions of the polymers will guide the rational design of new hydrogel systems. This knowledge enables specific modification (such as the incorporation of functional groups, adjustment of crosslinking density, or combination with other materials) that can fine-tune the hydrogel’s activity and performance according to the desired biological response.

Properties such as surface charge, roughness, topography, porosity, and controlled degradability directly influence how immune, epithelial, and connective tissue cells recognize and integrate with the matrix. Achieving an optimal balance between antimicrobial activity and biocompatibility will enable the development of systems that not only inhibit microbial growth but also promote tissue regeneration, modulate inflammation, and support orderly wound healing.

Thus, the study of polymer–cell interactions and the ability to chemically tailor polymeric matrices emerge as key pillars for optimizing biological performance and advancing toward truly functional, safe, and clinically relevant hydrogels—capable of actively contributing to antimicrobial resistance control and the future of regenerative medicine.

## 6. Future Perspectives

One of the most critical drawbacks of smart hydrogels is their often sluggish response rate. This slow response results from the slow diffusion of analytes into the gel matrix, which can compromise sensitivity and prolong detection times. Furthermore, conventional hydrogels are inherently mechanically weak and brittle. The lack of homogeneity in the cross-linking density (spatial inhomogeneity) within the polymer network maintains this macroscopic mechanical deficiency. The limited mechanical strength of certain hydrogels, such as those based on poly(N-isopropylacrylamide) (PNIPAAm), exemplifies this restricted efficacy. A key issue limiting the clinical viability of hydrogels is the uncertainty surrounding their long-term biosecurity and in vivo biodegradability. While synthetic polymer-based hydrogels often exhibit longer durability and better mechanical properties, they can be cytotoxic. The necessity of chemical cross-linking agents (e.g., photo-initiators like Irgacure 2959 (I2959) or N,N′-methylenebisacrylamide (MBAAm)) carries a risk of biotoxicity. Specifically, the formation of free radicals during photo-polymerization can induce cell damage. The incorporation of magnetic nanoparticles (MNPs) or photothermal agents (PTAs) while enhancing responsiveness and function, raises concerns about cellular damage and inflammation. The overly complex design of many advanced smart hydrogel systems complicates the assurance of long-term biocompatibility and safety. Moreover, complex preparation methods and high associated costs hinder their large-scale industrial production. Despite numerous innovative scientific achievements and publications, very few smart hydrogel solutions have successfully advanced to clinical trials. The effective use of stimuli-responsive hydrogels is currently impeded by the absence of clear regulatory guidelines and established standards.

To fully realize the potential of smart hydrogels, future research must concentrate on critical areas that systematically address these existing limitations, focusing on enhancing functionality, precision, and clinical relevance. The transition of smart hydrogels into clinical practice faces a major bottleneck, primarily due to the difficulty in translating promising results obtained in animal models to human physiological settings. Although extensive research exists, including studies on injectable PNIPAAm (Poly(N-isopropylacrylamide)) based materials, only a limited number of smart hydrogels have successfully achieved practical clinical use. For instance, despite favorable outcomes reported in diabetic dog and minipig models for glucose-responsive insulin delivery systems, successful assessment in human clinical trials has yet to be achieved. This disparity may stem from the insufficient understanding of quantitative interspecies differences, making the prediction of clinical outcomes in humans challenging. Rigorous and long-term clinical studies and comprehensive in vivo research are mandatory to evaluate the long-term safety and efficacy of surface-modified hydrogels. It is anticipated that the response of hydrogels to stimuli in vivo may differ from observations made in vitro. Individual patient variations and disease progression (e.g., cancer stages) can affect the repeatability of the smart hydrogel’s response in a clinical context. While percutaneous injection of injectable hydrogels is technically simple, targeting the precise defect site in unseen subcutaneous tissue poses difficulties in correctly positioning the hydrogel, particularly in applications like regenerative medicine.

Future efforts should prioritize the synthesis of programmable smart hydrogels capable of responding to multiple stimuli (multi-responsiveness) simultaneously. Since disease microenvironments (e.g., low pH, high temperatures, specific enzyme concentrations, and ROS levels) are complex, these matrices must integrate synergistic effects (e.g., drug delivery, embolization, and hyperthermia) to deliver several therapeutic modalities effectively. AI and ML are emerging as critical tools for enhancing smart hydrogel design and optimization. AI-driven modeling could enable the creation of hydrogels that adapt to the unique physiological factors of each patient, paving the way for personalized medicine. Such integration will accelerate the understanding of nanoscale phenomena and provide advanced tools for high-throughput nanomaterials research. Future studies must focus on precisely regulating the release kinetics of therapeutic agents (such as drugs and proteins) from hydrogels and designing systems with highly sensitive and reliable release profiles. Research should also advance the creation of sophisticated nanostructured hydrogels, such as nanocomposite (NC) hydrogels and multi-gradient self-assembled monolayers (SAMs), to tackle complex biological challenges, including promoting the spatially regulated differentiation of stem cells. Expanding the scope of smart hydrogel applications necessitates further improvements in fast response kinetics, superior mechanical characteristics (e.g., Young’s modulus, toughness, and fracture strength), and robust validation of biocompatibility.

## Figures and Tables

**Table 1:** Classification of Smart Hydrogels Based on Stimulus Type.

Stimulus Type	Basic Mechanism	Typical Polymer/Material Examples	Result/Impact (Response Type)
**Physical**	• A shift in the balance between hydrophilic/hydrophobic groups in polymer chains triggers a volumetric phase transition at the critical solution temperature (LCST/UCST). • Isomerization of photosensitive groups (e.g., azobenzene) or initiation of structural/chemical changes through photothermal effects (localized heat). • Osmotic pressure differences are formed as a result of the movement of mobile ions in ionic polymer networks.	Poly(N-isopropyl acrylamide) (PNIPAAm) and its derivatives (LCST), poly(vinyl methyl ether) (PVME), poly(ethyleneglycol) (PEG)-Polyester block copolymers (e.g., PEO-PPO-PEO)	• Sudden and reversible volume change (swelling/shrinking). Sol–gel phase transition. Shape change (bending/stretching), actuator or artificial muscle function. • Sudden and reversible volume change (swelling/shrinking), sol–gel phase transition, bending/movement (actuators). Osmotic pressure difference due to ion movement in ionic polymers in an electric field. Isomerization in the presence of light (e.g., azobenzene) or photothermal heating.
**Chemical**	• Proton acceptance/donation by ionizable groups (carboxyl, amine, etc.) in polymer chains. This changes the electrostatic repulsion force and osmotic pressure. • At high ion concentrations, the shielding of the electrostatic repulsion between ionic groups leads to a reduction in swelling. • Changes in polymer structure due to oxidation/reduction reactions of redox-active bonds (e.g., disulfide)	Poly(acrylic acid) (PAAc), Poly(methacrylic acid) (PMAAc) (anionic); chitosan, Poly(N,N′-dimethylaminoethyl methacrylate) (PDMAEMA) (cationic), Polymers containing disulfide (S-S) or diselenide bonds	• Continuous or sudden (sharp) change in volume and permeability depending on the degree of ionization. The change in electrostatic repulsion forces is the main driving force in polyelectrolyte gels.
**Biological**	• Change or disruption of cross-link density by enzymatic hydrolysis of enzyme-sensitive binding sites (e.g., peptide sequences) in the polymer network. • Motifs such as glucose oxidase (GOx) or phenylboronic acid (PBA) sense glucose concentration, triggering volume changes and insulin release, usually through pH or cross-link changes. • Specific antigen–antibody binding controls swelling or shrinkage by forming physical cross-links.	PEG-based hydrogels containing peptide sequences sensitive to matrix metalloproteinases (MMPs), Glucose Oxidase (GOx), and pH-sensitive polymers (e.g., PAAc), or phenylboronic acid (PBA) groups.	• Targeted and controllable drug release by mimicking specific biological signals. Biosensor applications (e.g., glucose level monitoring). Cross-link density reduction/disruption and drug release in the presence of enzymes. Volume/phase transition in response to the biomolecule (e.g., pH decrease and swelling in response to glucose).

**Table 2:** Advantages and disadvantages of stimulus-responsive smart hydrogel types [47,49,51,61,71,72,73,74].

Smart Hydrogel Type	Advantages	Disadvantages/ Challenges
Temperature Sensitive (TRHs)	They can have LCST values close to body temperature (32–37 °C) for in situ gelation, making them ideal for injectable systems. Thermosensitive sol–gel transformations can be reversible or irreversible. Simple triggering; works with body temperature.	Injectable systems have the potential for needle blockages, acidic degradation products, and burst release. Limited mechanical strength
pH Sensitive (PRHs)	Responds to local pH changes at pathological sites such as tumors, infection sites, or the gastrointestinal tract, enabling targeted release. Among chemical stimuli, it may be more challenging to change the pH within the system quickly and precisely than with other physical stimuli.	Affects the degree of ionization that triggers volume transition It requires high precision preparation.
Light Sensitive (LRHs)	Light application can be performed instantly and with high accuracy compared to other systems. Remote and fast control Provides spatial and temporal control.	Three-dimensional printing light-responsive hydrogels is generally not easy. Limited tissue penetration Light penetration into deep tissues is limited unless near-infrared (NIR) light is used.
Magnetically Responsive (MHRs)	When applied, magnetic fields offer remote, non-invasive control and local heating (hyperthermia) via superparamagnetic nanoparticles. It has excellent potential to penetrate deep organs and be directed to the targeted area.	Toxicity and Biocompatibility Issues
Electrically Sensitive (ERHs)	It can function as an actuator (artificial muscle) by converting electrical energy directly into mechanical work. It can increase the drug release rate proportionately to the applied electric current and provide pulsative release control.	The mechanical force (motion) on polymer-grafted surfaces is generally small/microscopic. They can be time-consuming and expensive to prepare.
Enzyme Sensitive	It responds with high specificity to pathological conditions such as the tumor microenvironment. It enables precise and accurate drug release (fixed point/fixed time/fixed amount).	In pathological conditions, the distribution and concentration of stimuli (enzymes, etc.) may change unpredictably.
Redox Sensitive	It can dynamically tune mechanical properties (e.g., transition between soft and hard states via Fe^3+^, Fe^2+^ transformation).	Slow response time
Sensitive to Ionic intensity	It can be adjusted to optimize water retention and nutrient delivery in high salinity agricultural soils.	High ion concentrations reduce swelling by masking electrostatic interactions.
Pressure/Mechanical Sensitive	It can trigger drug release in response to mechanical stimuli such as stretch or shear force (stretch-triggered release).	Poor Mechanical Properties

**Table 3:** Bibliographic distribution of Scopus-indexed publications on stimuli-responsive hydrogels with antibacterial activity (2017–2025).

Search Query	Number of Documents	% of Total Stimuli-Responsive Antibacterial Hydrogels
Stimuli-responsive antibacterial hydrogels	12,503	100
Alginate- based Stimuli-responsive hydrogels	5137	41
Chitosan- based Stimuli-responsive hydrogels	8162	65
Other natural/synthetic-based stimuli-responsive antibacterial hydrogels	745	6.0

**Table 4:** Representative studies on alginate-based hydrogels for antimicrobial applications (2017–2025).

Refs.	Material/Design	Incorporated Agent	Microorganisms Evaluated	Experimental Model	Highlights
[88]	Supramolecular hydrogel of cationic peptides reinforced with alginate	Cationic peptides (AMP-like)	*E. coli*	In vitro	Alginate improves mechanical stability and rheological properties.
[89]	Oxidized hydrogels (polymer-CHO) crosslinked with aminoglycosides (neomycin, tobramycin, amikacin, among others)	Aminoglycosides (as part of the network)	*E. coli*, *S. aureus* (USA300), *S. epidermidis*, *P. aeruginosa* (PAO1)	In vitro and in vivo (mice)	Broad-spectrum antibacterial activity Release through network erosion Network properties depend on the number of amino groups in the antibiotic, oxidation degree, and polymer type.
[90]	Alginate-phenylboronic acid (ALG-BA) hydrogel with hyaluronic acid-cholesterol (HA-CH) micelles	Amikacin + Naproxen (in HA-CH micelles)	*S. aureus*, *P. aeruginosa*	In vitro and in vivo (infected rat wound)	Combined antibacterial and anti-inflammatory therapy Accelerated wound healing and inflammation modulation.
[91]	Carboxymethyl chitosan (CMCS)/Alginate hydrogel with sinoporphyrin (DVDMS) and PLGA nanoparticles loaded with bFGF	DVDMS + bFGF	MDR-*S. aureus*	In vitro and in vivo (infected burns)	99.99% eradication of MDR-*S. aureus* Promotes angiogenesis and accelerated tissue repair.
[92]	Alginate hydrogel with PDMC and polydopamine (PDA)	PDA	*S. aureus*, *E. coli*	In vitro and in vivo (infected rat wounds)	Antibacterial activity via photothermal effect under NIR irradiation Nearly complete wound closure (96–100% in 14 days) Pressure/temperature sensing capability.
[93]	PLGA/Alginate/reduced graphene oxide (r-GO) hydrogel in neem oil emulsion	Hesperidin	*S. epidermidis*, *C. albicans*	In vitro and in vivo (rat otitis media)	Antimicrobial and antifungal activity; biofilm inhibition; antioxidant and anti-inflammatory effects.

**Table 5:** Representative studies on chitosan-based hydrogels for antimicrobial applications (2018–2025).

Refs.	Material/Design	Incorporated Agent	Microorganisms Evaluated	Experimental Model	Highlights
[101]	Injectable conductive hydrogel of chitosan (CS), polyaniline (CP), and oxidized dextran (OD) as crosslinking agent	Ibuprofen and amoxicillin	*S. aureus*, *E. coli*	Antibacterial activity in vitro. Gelation and biocompatibility assays performed in vivo (rats).	Hydrogels exhibited antibacterial activity against *S. aureus* and *E. coli*. Enabled controlled and localized drug release in response to dual stimuli (electric field and pH). Hydrogels were biocompatible and biodegradable, as confirmed in vivo.
[102]	Hybrid hydrogel of chitosan (CS), bromothymol blue (BTB), and conjugated polymer (PTDBD)	Bromothymol blue (BTB) and conjugated polymer PTDBD	*Staphylococcus aureus*, *Klebsiella pneumoniae*, ampicillin-resistant *E. coli*	In vitro (biofilms, cultures) and in vivo (rats)	In situ visual diagnosis of infection through color change. Antibacterial photothermal activity under NIR irradiation. Effective system against *S. aureus* biofilms in infected wounds.
[103]	Adhesive hydrogel of quaternized chitosan (QSC), ferric ions (Fe), and protocatechualdehyde (PA)	No exogenous antibiotic; intrinsic antibacterial activity of chitosan; NIR-responsive	*S. aureus* MRSA, *E. coli*	In vitro/In vivo (infected wounds in rats)	Hydrogels displayed injectability, self-healing, excellent biocompatibility, antibacterial and antioxidant activities, and on-demand removability. Demonstrated mechanical strength, tissue adhesion, and wound healing ability.
[104]	Composite hydrogel of polyacrylate (PAA), quaternized chitosan (HACC), and ZnO nanoparticles	Mupirocin	*S. aureus* and *E. coli*	In vitro	Hydrogel showed mechanical strength, water absorption, and gas permeability. Biocompatible. Mupirocin was selectively released under inflammatory (alkaline pH) conditions. Antibacterial properties attributed to ZnO.
[105]	Carboxymethyl chitosan (CMCS)/hydroxyethyl cellulose (HEC) hydrogel	Polydiacetylene–ZnO nanosheets	*Escherichia coli* 25922	In vitro	pH-dependent water absorption capacity. Colorimetric response to pH changes. Antimicrobial activity attributed to ZnO release.
[106]	Polivinil alcohol (PVA)/chitosan/gelatin hydrogels	*Zataria multiflora* essential oil	*Candida albicans*	In vitro	Hydrogels exhibited antifungal activity and biocompatibility. Thermoresponsive behavior enabled compound release at body temperature
[107]	Sandwich-type CABP hydrogel: – Top layer: agarose/chitosan(CS)/BTB hydrogel – Middle layer: non-woven CS structure (NF/CS) – Bottom layer: agarose/CS/phthalocyanine (AG/CS/Pc)	Phthalocyanine (Pc, photosensitizer)	*S. aureus*	In vitro (biofilm)/In vivo (infected wounds in rats)	Enabled real-time detection of infection through color change in BTB in the top layer. Photosensitizer Pc enabled elimination of *S. aureus* with 99.9% inhibition under PDT.
[108]	Dual pH- and temperature-responsive hydrogel of carboxymethyl chitosan and Poloxamer 407	Naringenin + Ferulic acid (NAR-FA)	*Bacillus subtilis*, *E. coli*	In vitro (cytotoxicity, antimicrobial assays)/In vivo (diabetic rat ulcers)	Antimicrobial activity against *B. subtilis* and *E. coli*. Promoted re-epithelialization and reduced inflammation in diabetic wounds.
[23]	Chitosan–polivil pirolidona (PVP) hydrogel	Zinc MOFs and silver nanoparticles.	*S. aureus*, *E. coli*, *Fusarium solani.*	In vitro antimicrobial and wound healing assays.	Antimicrobial and antifungal activity. Non-cytotoxic; supported cell proliferation. Promoted wound healing in rat models.
[109]	Dual photothermal hydrogel of carboxymethyl chitosan methacrylate (CMCS), silk fibroin (SF), and bioactive glass (BG) for bone regeneration	Bioactive glass	*S. aureus*, *Porphyromonas gingivalis*	In vitro/In vivo (rat cranial defect model)	Injectable hydrogel with osteogenic and angiogenic properties. Antibacterial activity comparable to commercial collagen membranes.
[110]	Thermoresponsive hydrogel of poly(N-iso propylacrylamide) PNIPAM/Poloxamer 407 with pH-sensitive chitosan (CS)	Silver nanoparticles (AgNPs) + caffeic acid phenethyl ester + quercetin in nanolipid complexes (CQ-ML)	*Porphyromonas gingivalis*	In vitro/In vivo (rat model)	Trimodal therapy: (i) antibacterial (AgNPs) against periodontal biofilm; (ii) anti-inflammatory effect; (iii) alveolar bone regeneration. Injectable, in situ, pH-sensitive hydrogel with local microenvironment control and improved periodontitis outcomes.
[111]	Smart gelatin–CS hydrogel coated with polydopamine (pDA)	Quaternized chitosan (QCS) or poly(methacrylic acid) (PMAA)	*S. aureus* MRSA	In vitro/In vivo (infected wounds in rats)	Long-lasting antimicrobial activity with promotion of wound healing via fibroblast migration, angiogenesis, and collagen synthesis. Under physiological conditions, hydrated PMAA prevented bacterial adhesion. In acidic microenvironments, PMAA collapsed, exposing QCS with bactericidal activity.

## Data Availability

No new data were created or analyzed in this study.
